# Cardiovascular disease risk factors in chronic kidney disease: A systematic review and meta-analysis

**DOI:** 10.1371/journal.pone.0192895

**Published:** 2018-03-21

**Authors:** Rupert W. Major, Mark R. I. Cheng, Robert A. Grant, Saran Shantikumar, Gang Xu, Issaam Oozeerally, Nigel J. Brunskill, Laura J. Gray

**Affiliations:** 1 Department of Health Sciences, University of Leicester, Leicester, United Kingdom; 2 John Walls Renal Unit, Leicester General Hospital, University Hospitals of Leicester, Leicester, United Kingdom; 3 Department of Medical Education, University of Leicester, Leicester, United Kingdom; 4 Department of Infection, Immunity and Inflammation, University of Leicester, Leicester, United Kingdom; Universita degli Studi di Perugia, ITALY

## Abstract

**Background and objectives:**

Chronic kidney disease (CKD) is a global health burden and is independently associated with increased cardiovascular disease risk. Assessment of cardiovascular risk in the general population using prognostic models based on routinely collected risk factors is embedded in clinical practice. In CKD, prognostic models may misrepresent risk due to the interplay of traditional atherosclerotic and non-traditional risk factors. This systematic review’s aim was to identify routinely collected risk factors for inclusion in a CKD-specific cardiovascular prognostic model.

**Design, setting, participants and measurements:**

Systematic review and meta-analysis of observational cohort studies and randomized controlled trials. Studies identified from MEDLINE and Embase searches using a pre-defined and registered protocol (PROSPERO ID—2016:CRD42016036187). The main inclusion criteria were individuals ≥18 years of age with non-endstage CKD. Routinely collected risk factors where multi-variable adjustment for established cardiovascular risk factors had occurred were extracted. The primary outcome was fatal and non-fatal cardiovascular events.

**Results:**

The review of 3,232, abstracts identified 29 routinely collected risk factors of which 20 were presented in more than 1 cohort. 21 cohorts were identified in relation to 27,465 individuals and 100,838 person-years. In addition to established traditional general population cardiovascular risk factors, left ventricular hypertrophy, serum albumin, phosphate, urate and hemoglobin were all found to be statistically significant in their association with future cardiovascular events.

**Conclusions:**

These non-traditional risk factors should be assessed in the development of future cardiovascular prognostic models for use in individuals with CKD.

## Introduction

Chronic kidney disease (CKD) is a global health burden estimated to affect up to 15% of adult populations [1–3] and is independently associated with increased cardiovascular (CV) disease risk similar to the risk of diabetes mellitus or coronary heart disease [1–2]. This risk increases as CKD advances and is evidenced by worsening excretory function, usually manifest as declining glomerular filtration rate, and increasing proteinuria [3–4]. The overall cost of CKD accounts for 1.3% of healthcare budgets [5] of which 13% is related to the excess myocardial infarctions and strokes associated with CKD [5].

Assessment of CV risk using prognostic models in the general population, particularly for primary prevention, is embedded in clinical practice [6–9]. Such prognostic models use data from routinely collected risk factors and can be automated using electronic medical records into routine clinical care. CV prognostic models developed specifically for CKD have significant methodological weaknesses, including no external validation and limited model metrics’ assessment, and thus may miscalculate risk in CKD. This contributes to their lack of clinical utility [10].

To our knowledge no systematic review has been performed to identify routinely collected risk factors that may potentially contribute to a composite CV outcome prognostic model in CKD. A new risk factor is only clinically useful if it adds predictive performance to a model beyond currently utilized standard risk factors, i.e. once a model has been adjusted for said factors, therefore additional risk factors must be novel and routinely collected in clinical care. Therefore, assessment of these factors is crucial before prognostic models can be rationally optimised.

Specific validation in CKD is warranted because the relative role of atherosclerosis in CV outcomes diminishes, and is replaced by the confounding—‘non-traditional’ CV risk factors. These uremia-related risk factors may have an increasingly important role with advancing CKD [11]. This may warrant inclusion of risk factors such as calcium and phosphate [12], related to arteriosclerosis and reduced vascular compliance, in CKD-specific CV prognostic models. Equally, consideration of risk factors associated with cardiomyopathy, such as echocardiographic evidence of left ventricular dysfunction or systemic inflammation may also be justified [11]. Thus other novel routinely collected risk factors require consideration for validation of CV prognostic models in CKD.

The aim of this systematic review was to identify routinely collected risk factors with potential value in CV risk prediction in CKD beyond those already included in existing CV prognostic models to inform the development of future CKD-specific CV prognostic models.

## Methods

Ovid MEDLINE and Embase were searched using a pre-defined and registered systematic review and meta-analysis protocol [13] (PROSPERO ID—2016:CRD42016036187). Search strategies are available in the Supporting Information (Tables A and B in S1 File). Reporting of the current systematic review follows the PRISMA guidance, also available in the Supporting Information (S2 File). The inclusion criteria were observational cohort studies and secondary analyses of randomized controlled trials in adult (≥18 years of age) with either CKD stage 3a or worse (any eGFR formula <60 ml/min/1.73m^2^) or proteinuria based on standard definitions [14]. The search was limited to English language manuscripts. General population studies with subgroup analysis presenting results for CKD groups were also included. Studies including individuals with end-stage renal disease, either receiving maintenance dialysis or with a renal transplant, were excluded. Studies of outcomes after acute kidney injury were also excluded. The minimum follow-period was six months. A formal definition of CKD using a standardised eGFR formula was first established in 1999 [15], therefore the search range was restricted from this date until 20^th^ October 2017.

The primary outcome was a composite of CV disease events which includes acute coronary syndrome (including unstable angina), congestive cardiac failure and ischemic stroke. Composite CV outcomes including CV-specific mortality were included unless CV events were grouped with all-cause mortality and/or renal related outcomes.

For the purposes of this paper ‘risk factor’ will be used throughout to mean a measurable variable at the start of a study that is associated with a future CV disease event during the study’s follow-up. Any variable was considered as a candidate risk factor if it was collected at or prior to the start point of the observational period for the study. In addition, factors were only included if they were likely to be routinely collected as part of standard primary care clinical practice. Whether a variable was routinely collected was assessed independently by three clinicians (RM, IO, GX). Where there was disagreement regarding a variable’s inclusion, it was discussed between the three assessors until a consensus was reached. For all other stages of the methods, assessment was performed independently by at least two of the authors. Where discrepancies occurred, results were compared until a consensus was reached. If no consensus was achievable, a further author was consulted to make a final decision.

The title and abstracts of all studies identified by the literature search were assessed. The full text of any abstract meeting the inclusion criteria was then reviewed. Data were extracted using a standardised extraction form which included a risk of bias assessments based on the ‘Quality in Prognostic Studies’ tool [16]. Confounders adjusted for in each model were also extracted. The data extraction form was modified and optimised after data collection from three manuscripts had been performed. High risk of bias was not used as a reason for excluding a study. Where missing data in relation to a cohort’s characteristics or model were not published, the corresponding author for the cohort was contacted via email.

Data for the risk factors were extracted in the form of hazard ratios (HR) and 95% confidence intervals (CI) for the primary outcome. Categorical risk factors were standardised to the same reference category and continuous variables to the same units (Table C in S1 File). For example, the gender risk factor was presented as the risk for being male. Where different units were reported for the same variable, those units reported in the majority of studies were used, and the minority studies’ results were converted to the same units. A random effects model using the Mantel-Haenszel method was used as heterogeneity was expected to be present [17]. Data were meta-analysed where more than one study reported results for the same risk factor. Heterogeneity was assessed using the I^2^ statistics. Subgroup analysis was considered by CKD stage including both eGFR and proteinuria. Due to the limited clinical applicability and bias of univariate analysis of risk factors, only results from studies where multi-variate adjustment for traditional CV risk factors were considered further. Models were then assessed for the number of ‘core’ risk factors they adjusted for. Core risk factors included age, gender, ethnicity, body mass index, smoking, diabetes mellitus, hypertension, CV disease and dyslipidemia. These risk factors are all included in general population prognostic tools or have a firmly established association with CV disease risk [2,6,7,18]. In addition, because of their additive benefit to CV prognostic tools [4], eGFR and proteinuria measurements were also included as core adjustment co-variates. Where the same study had published results for a risk factor in more than one manuscript the paper with the most complete data was used. If the data were the same, the results from the most recent publication were used. Where more than one model was presented in the same publication, the model with the greatest number of core risk factors included was used. All statistical analysis was performed using Stata version 14.1.

## Results

**Fig 1:**
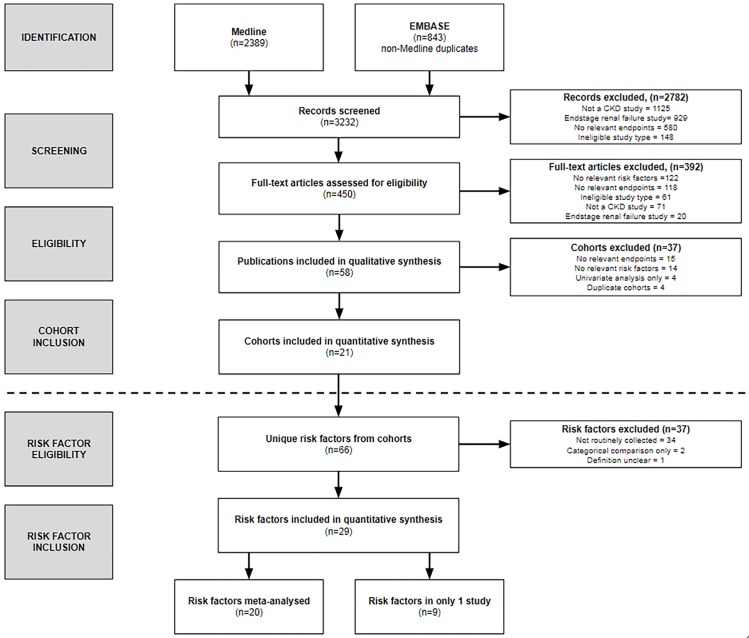
Flowchart showing the number of cohorts and risk factors identified, screened and included in the systematic review.

Three thousand two hundred and thirty-two abstracts were reviewed. Fig 1 shows the screening process, including the number of cohorts and risk factors identified, and reasons for any exclusion. Twenty-one cohorts were included in the systematic review [19–39]. Fourteen (66.7%) studies were observational cohort studies with recruitment from nephrology outpatient settings and the others were randomized controlled trials. Six cohorts provided additional data [19–24].

Overall a total of 27,465 individuals were included in these studies representing a cumulative total of 100,838 person-years. Table 1 summarises the characteristics of the cohorts contributing to the systematic review. The risk of bias for all studies was medium to high (see Table D in S1 File). In addition to the observational nature of the studies as a source of bias, other factors relating to study participant inclusion and exclusion, assessment of outcomes, reporting of missing data and statistical methods were considered. Six cohorts (28.6%) were recruited from a single-center. CV outcomes were broadly similar but 15 studies (71.4%) did not blind their outcome assessors. Seven cohorts (33.3%) reported no information in relation to missing data. No study pre-specified or registered their published analysis plan.

**Table 1:** Summary of 16 cohorts contributing data to systematic review.

Study Name	Publication Year	Journal	Study Type	Cohort Size	Mean/median follow-up (months)	Mean/median age, years	Male%	White%	Black%	Other ethnicity%	GFR Measurement	eGFR	urine	CVD%	DM%	HTN%
AASK[25]	2006	AJKD	RCT	1094	49	55	61.2	0	100	0	125-iothalamate	46	proteinuria 0.31mg/mg	51.6	0	100
Ankara[26]	2014	CJASN	Cohort	403	38	53.2	56.5	-	-	-	MDRD	~20% in each CKD category	1.61 g/day	13.4	22.6	15.9
CanPREDDICT[27]	2016	Kidney International	Cohort	2529	36	68.2	62.5	88.7	-	-	MDRD	28.0	ACR 16.3 mg/mmol	33.5^@^	48.2	26.5^$^
CARE FOR HOMe[19]	2014	CJASN	Cohort	444	31	65	60	99.8	-	0.2	MDRD	45+-16	proteinuria 37 mg/g	30.0	38	37.2^
CREATE[28]	2010	Current Medical Research & Opinion	RCT	291	24	59.9	48.8	-	-	-	CG	-	-	93.5	-	90.4
CRIC[29]	2013	AJKD	Cohort	3904	47	58.2	54.8	45.5	41.8	12.7	CRIC-GFR	44.8	1.07 g/day	33.4	48.5	86.1
CRISIS[30]	2015	Nephrology	Cohort	463	46	63.8	61.8	96	-	-	MDRD	29.4	0.49 g/L protein	29.4	31.3	13.0^$^
Digitalis[31]	2010	Circulation: Heart Failure	RCT	1974	57	68	65.6	89.2	-	10.8% 'non-white'	MDRD	47	-	100	50	60.2
Fujita[32]	2013	Heart and Vessels	Cohort	404	33	67	63.6	-	-	-	MDRD	24.1	351 mg/g Cr	33.2	37.6	73.5^
Genoa[33]	2016	CJASN	Cohort	445	71	64.1	62.0	100	0	0	MDRD	39.9	0.4 g/d	22.0	19.1	100
ICKD[20]	2013	CJASN	Cohort	3303	36	63.5	57.8	-	-	-	MDRD and EPI-CKD	23.4 (EPI-CKD)	PCR 1118.3 mg/g	26.4	44.6	67.1
Kaohsiung[34]	2013	Nephron Clinical Practice	Cohort	356	25	66.3	73	-	-	-	EPI-CKD	% stage given	dipstick	11.8	58.4	83.7
Kyushu[21]	2014	Hypertension Research	RCT	320	30	72	68.1	0	0	100% Japanese	Japanese equation	18.4	1.5 g/day	19.0	51	94
Leuven[22]	2015	Kidney International	Cohort	476	57	64	54.6	98.0	-	2.0% ‘non-Caucasian’	EPI-CKD	34	0.27 g/day	27.7	18.1	70.7^
Madrid[23]	2010	CJASN	RCT	113	23	71.6	64.6	100	0	0	MDRD	40.1	35.5 mg/d albuminuria	23.0	21	80^
MAURO[24]	2015	CJASN	Cohort	755	31	62	60	100	0	0	MDRD	36	0.6 milligram/24 hours	29.0	35	92
Naples[35]	2013	JACC	Cohort	436	57	65	58.3	100	0	0	MDRD	42.9	0.31g/day	30.5	36.5	72.9
OSERCE-2[36])	2015	CJASN	Cohort	742	35	66	65	99	0	1	MDRD	27.3	proteinuria 106 mg/g	11.0	66	94
Pravastatin[37]	2005	JASN	RCT	4670	64	62.3	21.3	>90	-	-	MDRD	56.7	dipstick	75.3	12.2	48.2
RRI[38]	2012	NDT	Cohort	305	32	59.5	50.5	78.4	17.7	3.9	MDRD,CG	28.2	ACR 192.0 (2–9259)	36.7	30.8	88.9
TREAT[39]	2016	Journal of Human Hypertension	RCT	4038	29	68	42.7	63.6	20.2	16.1	MDRD	33	PCR 0.39 g/g	36.5”	100	92.4

‘-‘ refers to data not presented.

^figure based on proportion on RAAS blocker, for the Madrid cohort also 29.2% on CCB and 63.7% on diuretics.

^$^refers to percentage with hypertensive nephropathy as cause of CKD.

“refers to number with coronary heart disease, 17.6% had cerebrovascular disease.

^@^refers to proportion with ischaemic heart disease.

Journals: AJKD—American Journal of Kidney Disease, CJASN—Clinical Journal of the American Society of Nephrology, JACC—Journal of the American College of Cardiology, JASN—Journal of the American Society of Nephrology.

GFR measurement: CG—Cockcroft-Gault, CKD-EPI—Chronic Kidney Disease Epidemiology Collaboration, MDRD—The Modification of Diet in Renal Disease.

Sixty-six potential risk factors for CV events were identified (Table E in S1 File). Twenty-nine of these were deemed to be routinely collected and were therefore included in the systematic review. Nine risk factors were only reported in one study and therefore the data on 20 risk factors reported in multiple studies were pooled to produce a single estimate. The confounders which were adjusted for in all the included models are shown in Table 2. Age was corrected for in 20 out of 21 models (95.2%) and was the most frequently adjusted for variable. Diabetes mellitus was corrected for in 17 out of 19 models (89.5%) making it the co-morbidity most frequently corrected for. Ethnicity was included in four models, five models had no published ethnicity data and eleven cohorts had a population with a single ethnicity making up more than 90% of the population. Seventeen (81.0%) studies corrected for eGFR and eleven (52.4%) for proteinuria. Three studies (14.3%) adjusted for all established core CV risk factors.

**Table 2:** Summary of inclusion of established CV risk factors in multi-variate models included in systematic review.

Study Name	Age	Gender	Ethnicity	DM	HTN	CVD	Lipids	BMI	Smoking	eGFR	Proteinuria	Total
AASK[25]	●	●	N/A	N/A	N/A		●			●	●	5
Ankara[26]	●	●		●	●				●	●		6
CARE FOR HOMe[19]	●	●	N/A	●		●				●	●	6
CanPREDDICT[27]	●			●	●	●				●		5
CREATE[39]	●	●		●	●	●						5
CRIC[29]	●	●	●	●	●	●^	●	●	●	●	●	11
CRISIS[30]	●	●	N/A	●	●	●			●	●*		6
Digitalis[31]	●	●	●	●	●	N/A		●				6
Fujita[32]	●	●		●		●				●	●	6
Genoa[33]	●	●	N/A	●	●	●	●			●	●	8
ICKD[20]	●	●		●	●	●	●	●	●	●	●	10
Kaohsiung[34]				●	●	●				●		4
Kyushu[21]	●		N/A		●	●		●		●		5
Leuven[22]	●	●	N/A		●	●				●	●	6
Madrid[23]	●		N/A	●		●				●		4
MAURO[24]	●	●	N/A	●	●		●	●	●	●	●	9
Naples[35]	●	●	N/A	●	●	●		●		●	●	8
OSERCE-2[36]	●		N/A	●	●	●	●		●	●		7
Pravastatin[37]	●		N/A	●	●	●	●		●			6
RRI[38]	●	●	●	●	●	●	●	●	●	●	●	11
TREAT[39]	●	●	●	N/A		●					●	5
**Total**	**95.2%**	**71.4%**	**40.0%**	**89.5%**	**80.0%**	**85.0%**	**38.1%**	**33.3%**	**38.1%**	**81.0%**	**52.4%**	

‘Lipids’ includes correction for using any measure of serum lipids and/or use of lipid lowering medications. N/A indicates that the model could not include the variable because 100% of study individuals were in this category, for example AASK-RCT was a study of 100% African Americans with hypertension. Where this occurred the variable was not included for percentage calculations.

*corrected for serum creatinine.

**Fig 2:**
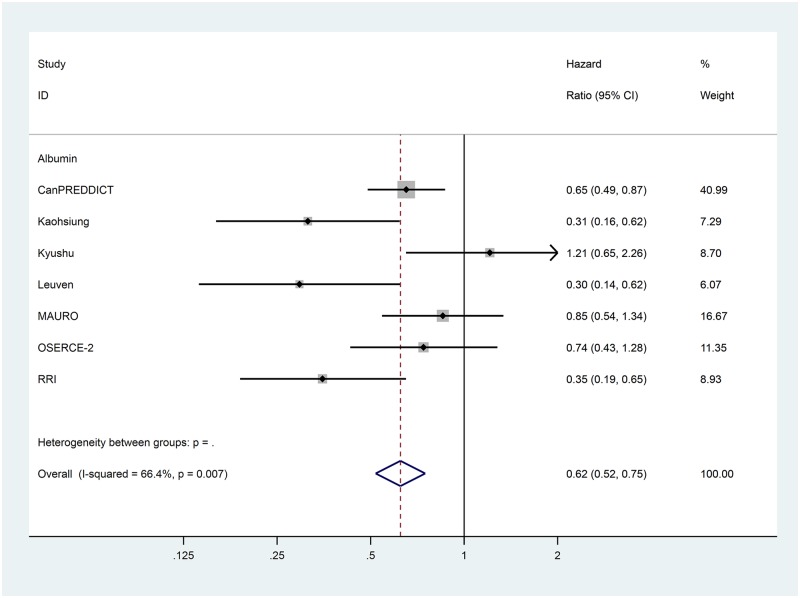
Forest plot for cardiovascular events of pooled hazard ratio for albumin per g/dL.

**Fig 3:**
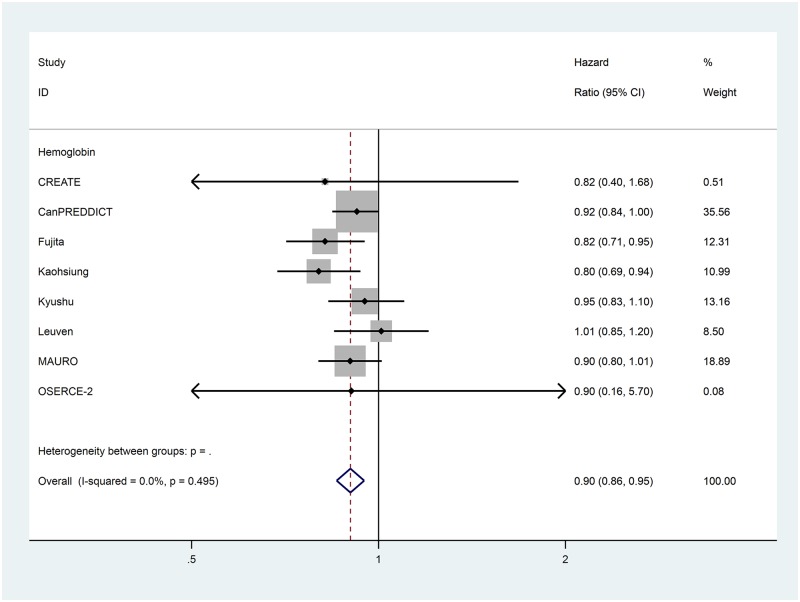
Forest plot for cardiovascular events of pooled hazard ratio for hemoglobin per g/dL.

**Fig 4:**
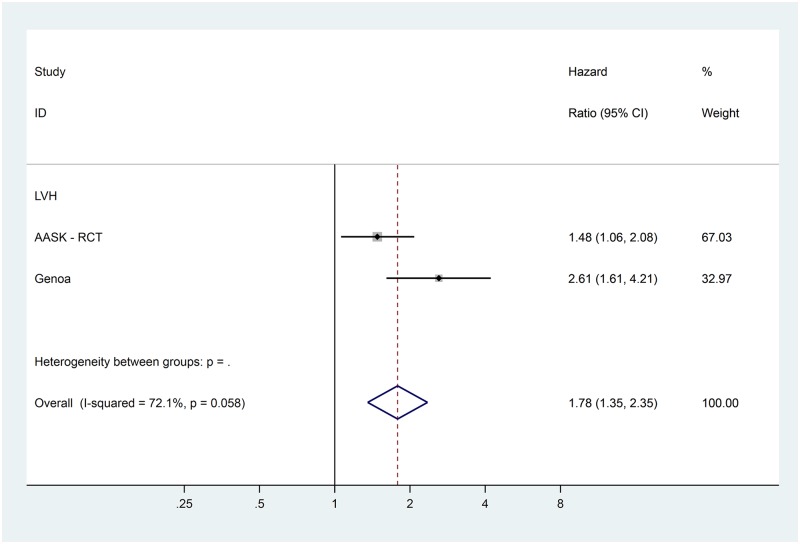
Forest plot for cardiovascular events of pooled hazard ratio for left ventricular hypertrophy.

**Fig 5:**
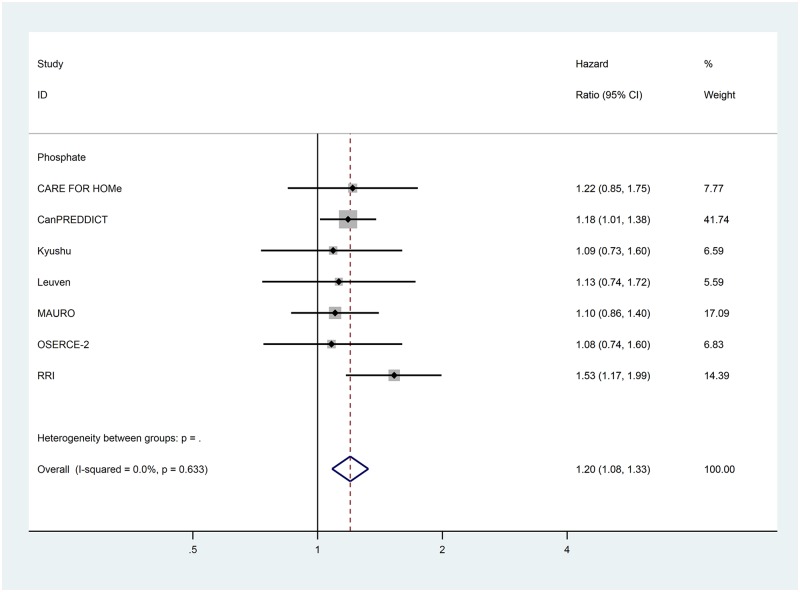
Forest plot for cardiovascular events of pooled hazard ratio for phosphate per mg/dL.

**Fig 6:**
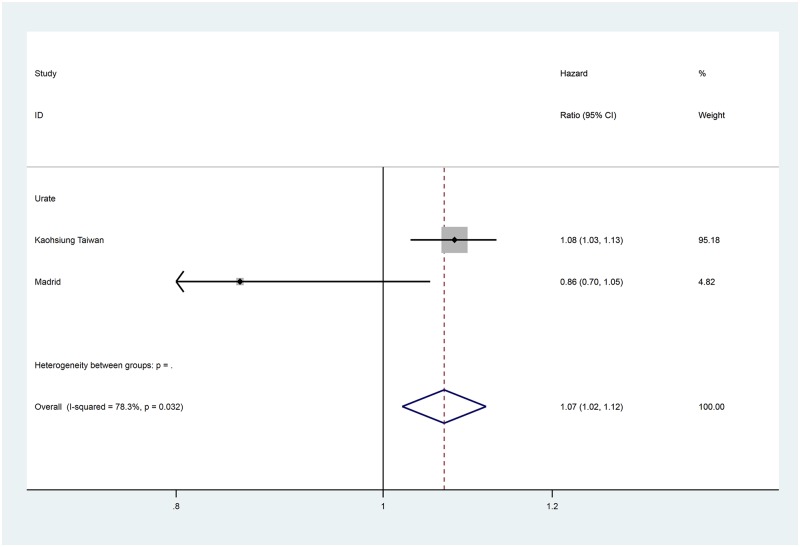
Forest plot for cardiovascular events of pooled hazard ratio for the urate per mg/dL.

Data for the extracted risk factors are shown in Table 3. The forest plots for the non-traditional risk factors of albumin, haemoglobin, phosphate and urate are shown in Figs 2 to 6 and forest plots for all other risk factors are shown in Figures A to N in S1 File. Within the traditional risk factors, male gender, increasing age, smoking, established CV disease, diabetes mellitus and increasing total cholesterol were all associated with statistically significant increased risk of a CV event. Systolic and diastolic blood pressures were not associated with increased CV event risk.

**Table 3:** Results for routinely collected risk factors for combined CV events.

Variable	Units (continuous)/ Comparator (categorical)	Number of Studies	Pooled HR	95% Confidence Interval	p-value for HR	I^2^ (%)
Male	female	9	1.451	1.220–1.726	<0.001	0.0
Age	per year	12	1.031	1.025–1.038	<0.001	58.6
Smoker	non-smoker	5	1.433	1.149–1.787	0.001	3.3
Body mass index	per kg/m^2^	3	0.994	0.964–1.025	0.7	23.0
Cardiovascular disease	no previous cardiovascular disease event	11	2.391	2.061–2.773	<0.001	68.1
Ischemic heart disease	no previous ischemic heart disease event	5	2.406	1.870–3.096	<0.001	43.2
Congestive heart failure	no diagnosis of congestive heart failure	3	1.325	0.989–1.774	0.06	0.0-
Peripheral vascular disease	no diagnosis of peripheral vascular disease	1	2.49	1.10–5.63	0.03	-
Diabetes mellitus	no diabetes mellitus	14	1.454	1.338–1.579	<0.001	73.5
Systolic blood pressure	per mmHg	8	1.002	0.999–1.004	0.17	77.8
Diastolic blood pressure	per mmHg	3	0.999	0.993–1.005	0.67	0.0
Mean arterial pressure	per 10 mmHg	1	1.14	1.03–1.27	0.01	-
Pulse pressure	per mmHg	3	1.002	0.998–1.005	0.38	58.7
Left ventricular hypertrophy	no left ventricular hypertrophy on echocardiogram	2	1.78	1.354–2.351	<0.001	72.1-
Pulmonary hypertension	no pulmonary hypertension on echocardiogram	1	1.23	1.00–1.52	0.04	-
Albumin	per g/dL	7	0.624	0.519–0.749	<0.001	66.4
Bicarbonate	per mEq/L	1	0.99	0.95–1.03	0.6	-
Cholesterol to HDL ratio	ratio	1	1.03	0.998–1.065	0.07	-
Calcium	per mg/dL	1	0.846	0.503–1.422	0.5	-
Hemoglobin	per g/dL	8	0.901	0.856–0.948	<0.001	0.0
HDL Cholesterol	per mg/dL	1	0.998	0.992–1.003	0.5	-
LDL Cholesterol	per mg/dL	2	1.001	0.999–1.003	0.2	0.0
Non-HDL Cholesterol	per mg/dL	2	1.001	1.000–1.003	0.04	70.4
Parathyroid hormone	per pg/mL	1	1.00	0.99–1.00	1.00	-
Phosphate	per mg/dL	7	1.198	1.084–1.325	<0.001	0.0
Sodium	per mmol/L	1	0.954	0.919–0.990	0.01	-
Total cholesterol	per mg/dL	3	1.001	1.000–1.002	0.01	65.8
Urate	per mg/dL	2	1.068	1.021–1.117	0.004	78.3
Urea nitrogen	per 5mg/dL	1	1.14	1.02–1.29	0.03	-

Abbreviations: HDL—high density lipoprotein, HR—hazard ratio, LDL—low density lipoprotein.

Results are given to 3 decimal places, unless data were only available from a single study that published results to 2 decimal places.

In the meta-analysis, non-traditional risk factors associated with increased risk of CV events were albumin (pooled HR 0.62 per g/dL increase, 95% CI 0.52–0.75, p<0.001), haemoglobin (pooled HR 0.90 per g/dL increase, 95% CI 0.86–0.95, p<0.001), phosphate (pooled HR 1.20 per mg/dL increase, 95% CI 1.08–1.33, p = 0.005) and urate (pooled HR 1.07 per mg/dL increase, 95% CI 1.02–1.12, p = 0.004). Left ventricular hypertrophy on echocardiogram (pooled HR 1.78, 95% CI 1.35–2.35, p<0.001) was also found to be associated with an increased risk of a CV event. Serum urea nitrogen, sodium and pulmonary hypertension on echocardiogram were all statistically significant but only present in one study each. Calcium, bicarbonate and parathyroid hormone were not associated with altered risk in the single studies in which they were included.

Heterogeneity varied substantially between variables (Table 3). Of the potential novel risk factors for incorporation in to prognostic models albumin (I^2^ = 66.4%), urate (I^2^ = 78.3%) and left ventricular hypertrophy (I^2^ = 72.1%) showed substantial levels of heterogeneity. Based on our pre-specified protocol, subgroup analyses to explore heterogeneity were considered for eGFR and proteinuria stages. These sub-analyses, and other *post hoc* analyses based on core cohort characteristics in Table 1, did not explain the heterogeneity for albumin. For urate and left ventricular hypertrophy, exploration of heterogeneity was limited by the inclusion of only two studies in the systematic review.

## Discussion

Whilst CV prognostic models are well established for the general population [6,7] it is unclear how well these models perform in patients with CKD [10]. CV prognostic models developed specifically for those with CKD exist but have poor methodology and limited clinical applicability [10]. The current systematic review, using a pre-defined and registered protocol [13], presents the association between routinely collected risk factors and CV disease events in individuals with CKD. The results confirm that most traditional atherosclerotic related risk factors confer risk in CKD populations. These include age, gender, smoking, established CV disease and diabetes mellitus, all of which were statistically significant risk factors that are incorporated in general population prognostic models and/or are established risk factors.

Studies of non-traditional risk factors associated with uremia-related arteriosclerosis and cardiomyopathy were also identified by the systematic review. Of these risk factors, albumin, haemoglobin and phosphate were included in at least four studies and had a statistically significant pooled hazard ratio for CV events. Other non-traditional risk factors that could be candidate risk factors for inclusion in a CV prognostic model include those associated with cardiomyopathy, such as left ventricular hypertrophy, urate, and those associated with both cardiomyopathy and arteriosclerosis including calcium, parathyroid hormone and urea nitrogen. Some of these risk factors have been considered in prognostic models identified by the previous systematic review of Tangri *et al* [10]. McMurray *et al* demonstrated an association of CV outcomes with serum albumin but not urea nitrogen [40].

The results of some risk factors were more difficult to interpret. Systolic and diastolic blood pressures were not statistically significant in their association with CV events. However, mean arterial pressure was in the single study in which it was considered. Previous studies, including individual participant meta-analysis, have suggested that the relationship of blood pressure with mortality and CV events in CKD is non-linear and may be due to uremic related myocardial and vascular remodelling [41–43]. The limited availability of study-level data, and therefore the opportunity to study non-linear relationships of blood pressure to CV events in CKD, makes it difficult to draw a firm conclusion. The ‘Blood Pressure Lowering Treatment Trialists’ Collaboration’ identified that blood pressure lowering in CKD is probably beneficial but was unable to identify a clear target [44]. Recent analysis of the SPRINT trial in CKD suggested a possible reduction of CV events with more intensive systolic blood pressure control of <120mmHg versus <140mmHg (HR 0.81, 95% CI 0.63 to 1.05) [45]. Similarly, lipid measurements, including total cholesterol and low density lipoprotein cholesterol, did not have a clear relationship. A previous study of myocardial infarction events has suggested a weaker association with low density lipoprotein cholesterol as CKD advances [46]. Similarly, the association of body mass index with CV events was unclear. We were unable to assess the risk associated with ethnicity as most studies did not present data that could be utilised in models, often because ethnicity was completely, or nearly, homogenous.

Heterogeneity between studies limits the interpretation of the results of meta-analyses, particularly in observational studies [47–49]. Further, poor reporting of individual studies makes comparison of results difficult [50–51]. The ideal method for selecting and combining studies is uncertain, but by limiting our analysis to studies with at least some adjustment for traditional CV risk factors and CKD severity, we aimed to reduce heterogeneity but at the cost of reduced power, via exclusion of some cohort’s results, of the meta-analysis. This approach also ensures that the results of the reported risk factors reflect the additional prognostic information above already established risk factors. Whilst individual patient data meta-analysis is the ‘gold standard’, the additional data from six studies used in the current study may have reduced bias.

Despite this conservative approach, heterogeneity was substantial [17] for nine risk factors. Two characteristics of the cohorts and their analysis may explain this. Firstly, the difference in variable standardisation between studies’ models may contribute to heterogeneity. Secondly, cohorts varied in the typical stage of CKD, measured through both eGFR and proteinuria, represented and this may have further increased heterogeneity.

Further limitations include, the conversion of many prognostic factors from continuous to categorical variables, leading to a loss of statistical power and comparison difficulties between studies due to differing thresholds [52–55]. Thirdly, models often presented results to a limited number, typically two, decimal places. This was particularly an issue when a continuous variable such as age or blood pressure was presented. The results published would often be the same for both HR and 95% CI e.g. HR 1.01 (95% CI 1.00 to 1.01), thus when meta-analysed the calculation of the standard error was likely to be inaccurate. We avoided changing reported HR units where possible to reduce any further inaccuracies introduced through rounding. Finally, data for eleven risk factors were only included in one study each, of which four had statistically significant association with CV disease events. Therefore, replication of these findings for peripheral vascular disease, pulmonary hypertension, mean arterial pressure and serum urea nitrogen in other CKD populations is required.

The relatively small number of studies identified by the systematic review reflects its specific pre-specified inclusion criteria. This specificity relates to the outcome inclusion criteria of composite cardiovascular events including CV specific mortality but excluding all-cause mortality and renal related events. Prominent CKD related studies were identified by the literature review but excluded based on the inclusion criteria and/or the nature of the risk factors presented (Table D in S1 File).

Full guidance on presenting risk factor models has been published by the PROGRESS consortium [56]. We would therefore recommend for future studies of CV risk factors in CKD, models should aim to provide a rationale for the variables used for model adjustment and avoid categorisation of continuous variables.

Based on the findings of this systematic review, at a minimum, the development of CKD CV prognostic models should assess traditional and non-traditional CV risk factors including left ventricular hypertrophy, serum albumin, hemoglobin, phosphate, and urate.

## Supporting information

S1 File **Table A**—Medline Search Strategy
**Table B**—EMBASE Search Strategy
**Table C**—Standardization of Variables
**Table D**—Summary of bias assessment for included studies
**Table E**–List of all 66 Risk Factors identified
**Figures A to N**—Forest Plots for all Risk Factors Meta-analysed
(DOC)

S2 File PRISMA checklist. (DOC)
