# Supplementary material for: Cardiovascular disease risk factors in chronic kidney disease: A systematic review and meta-analysis
Source: PLoS One. 2018 Mar 21;13(3):e0192895. doi: 10.1371/journal.pone.0192895 (PMC5862400; doi:10.1371/journal.pone.0192895)
Supplement: S1 File — Table A—Medline Search Strategy Table B—EMBASE Search Strategy Table C—Standardization of Variables Table D—Summary of bias assessment for included studies Table E–List of all 66 Risk Factors identified Figures A to N—Forest Plots for all Risk Factors Meta-analysed (DOC) [file pone.0192895.s001.doc]

**Supporting Material for ‘Cardiovascular disease risk factors in chronic kidney disease: a systematic review and meta-analysis’**

Major RW, Cheng MRI, Grant RA, Shantikumar S, Xu G, Oozeerally I, Brunskill NJ, Gray LJ

**Supporting Table A** - Medline Search Strategy

**Supporting Table B** - EMBASE Search Strategy

**Supporting Table C** - Standardization of Variables

**Supporting Table D** - Summary of bias assessment for included studies

**Supporting Table E** – List of all 66 Risk Factors identified

**Supporting Figures A to N** – Forest Plots for all Risk Factors Meta-analysed

**Supporting Table A - Medline Search Strategy**

Database: Ovid MEDLINE(R) In-Process & Other Non-Indexed Citations and Ovid MEDLINE(R)

1946 to 20^th^ October 2017

Search Strategy:

--------------------------------------------------------------------------------

1 exp RENAL INSUFFICIENCY, CHRONIC/

2 "chronic renal".ti,ab.

3 "chronic kidney".ti,ab.

4 "ckd".ti,ab.

5 exp CARDIOVASCULAR DISEASES/

6 "cardi*".ti,ab.

7 "heart*".ti,ab.

8 "coronary*".ti,ab.

9 "myocard*".ti,ab.

10 "ischem*".ti,ab.

11 "ischaem*".ti,ab.

12 "stroke*".ti,ab.

13 "cerebrovasc*".ti,ab.

14 "cerebral vascular*".ti,ab.

15 "cohort*".ti,ab.

16 exp Cohort Studies/

17 "random*".ti,ab.

18 "rct*".ti,ab.

19 exp Randomized Controlled Trials as Topic/

20 "risk*".tw.

21 "prognosis*".tw.

22 "predict*".tw.

23 "associat*".tw.

24 "factor*".tw.

25 20 or 21 or 22 or 23 or 24

26 5 or 6 or 7 or 8 or 9 or 10 or 11 or 12 or 13 or 14

27 15 or 16 or 17 or 18 or 19

28 1 or 2 or 3 or 4

29 25 and 26 and 27 and 28

30 limit 29 to yr="1999 -Current"

31 limit 30 to (english language and humans and "all adult (19 plus years)")

32 limit 31 to (clinical study or clinical trial, all or clinical trial or meta analysis or observational study or pragmatic clinical trial or randomized controlled trial or systematic reviews or validation studies)

**Supporting Table B - EMBASE Search Strategy**

exp RENAL INSUFFICIENCY, CHRONIC/ OR "chronic renal".ti,ab. OR "chronic kidney".ti,ab. OR "ckd".ti,ab.

AND

exp CARDIOVASCULAR DISEASES/ OR "cardi*".ti,ab. OR "heart*".ti,ab. OR "coronary*".ti,ab. OR "myocard*".ti,ab. OR "ischem*".ti,ab. OR "ischaem*".ti,ab. OR "stroke*".ti,ab. OR "cerebrovasc*".ti,ab. OR "cerebral vascular*".ti,ab.

AND

"cohort*".ti,ab. OR exp Cohort Studies/ OR "random*".ti,ab. OR "rct*".ti,ab. OR exp Randomized Controlled Trials as Topic/

AND

"risk*".tw. OR "prognosis*".tw. OR "predict*".tw. OR "associat*".tw. OR "factor*".tw.

limit to yr="1999 -Current"

limit to (english language and humans and "all adult (19 plus years)")

limit to (clinical study or clinical trial, all or clinical trial or meta analysis orobservational study or pragmatic clinical trial or randomized controlled trial or systematic reviews or validation studies)

**Supporting Table C - Standardization of Variables**

Hazard ratios (HR), their associated upper (U) and lower (L) 95% confidence intervals (CI) and p-values were extracted. Where these were not available, event rates were used. There was no restriction on the number of risk factors that one study could supply to the analysis. Results for categorical variables were standardised to the same reference category. For example, the gender variable was presented as the risk for being male. Therefore if risk was presented for being female, data was converted as follows:

- HR: HR(male) = 1/HR(female)
- Upper 95% CI: UCI(male) = 1/LCI(female)
- Lower 95% CI: LCI(male) = 1/UCI(female)

In the case of continuous variables, units were standardised to the same unit. For example, to convert hemoglobin (Hb) from g/l to g/dl for HR and CIs:

- HR: HR(Hb_g/dl) = HR(Hb_g/l)^10
- Upper 95% CI: UCI(Hb_g/dl) = UCI(Hb_g/l)^10
- Lower 95% CI: LCI(Hb_g/dl) = LCI(Hb_g/l)^10

**Supporting Table D - Summary of bias assessment for included studies**

| **Study** | **Study participation** | **Study exclusion/attrition** | **Outcome measurement** | **Missing data** | **Statistical Analyses and Reporting** |
| --- | --- | --- | --- | --- | --- |
| AASK-RCT | African Americans with hypertensive nephrosclerosis and a GFR of 20–65 ml/min per 1.73 m^2^ recruited from February 1995 to September 1998. | 2802 screened, 1094 enrolled, 186 excluded for this analysis - due to incomplete BP measurements (168), deaths within first 12 months (15), 'censored' (3). If ESRD reached then patient also censored (153). | Cardiovascular death, cardiovascular revascularization, nonfatal myocardial infarction (MI), hospitalization for heart failure, or stroke. | 168 excluded due to incomplete BP data. Amlodipine arm terminated early September 2000. 7 lost to follow-up. 20 deaths after ESRD reached excluded. | Trial was a 3x2 factorial design with BP target and 1 of 3 possible anti-hypertensives. Cox proportional hazards regression for analysis included in current systematic review. |
| Ankara | Patients referred to outpatient renal unit between March 2006 to February 2011. | 1276 eligible, 873 excluded due to 'conditions that may influence endothelial dysfunction', 65 excluded due to acute infection or unwillingness to participate. | Fatal and non-fatal stroke and myocardial infarction. Recorded by telephone contacts and routine outpatient clinic visits. | 80 lost to follow-up, 73 withdrew consent. No other mention of missing data | Cox proportional hazards models. Does not report full multivariate model confounding factors. |
| CanPREDDICT | Multi-centre prospective cohort study. Recruitment dates not specified. Patients with CKD. eGFR between 15 to 45 ml/min/1.73m². | 2529 included in final analysis. Number screened not given in current paper. Exclusion criteria - organ transplant recipient, life expectancy less than 12 months, acute vasculitis | Ischemic cardiovascular events ‘were adjudicated and defined as myocardial infarction, unstable angina, ischemic stroke, coronary revascularization, new onset of coronary heart disease (proven by cardiac catheterization), amputation due to peripheral vascular disease, peripheral artery bypass, and gangrene.’ Unclear if assessors ‘a cardiologist, a nephrologist, and a neurologist’ were blinded to patient characteristics for this particular study. | 15 individuals excluded as TMAO results not available | Multivariable cox proportional hazards regression |
| CARE FOR HOMe | Single outpatient renal recruitment from September 2008 to November 2012. Patients with CKD stages 2-4, eGFR 15-89 ml/min per 1.73m2. Patients with CKD stage 2 had 'one or more markers of kidney damage, including albuminuria and/or plasma creatinine/cystatin C above references values'. | Systemic immunosuppressive medication and those with concomitant human immunodeficiency virus infection, clinical apparent infections (defined as CRP levels above 50 mg/l, and/or requiring systemic antibiotic therapy), active cancer disease, malignant hematological disorders, and/or acute renal failure (defined as increase of plasma creatinine >50% within four weeks) were excluded from study participation. Moreover, we excluded allograft recipients, pregnant women, and subjects <18 years of age' | Atherosclerotic events/death, which comprises acute myocardial infarction (defined as a rise in troponin T above the 99th percentile of the reference limit accompanied by symptoms of ischemia and/or electrocardiographic changes indicating new ischemia) , surgical or interventional coronary/cerebrovascular/peripheral arterial revascularization, stroke (defined as rapidly developing clinical symptoms or signs of focal [or at times global] disturbance of cerebral function lasting .24 hours [unless interrupted by surgery] or leading to death, with no apparent cause other than of vascular origin), amputation above the ankle'. | None lost to follow-up. | Multivariate Cox regression. |
| CREATE | 605 enrolled, 476 completed study (roughly equal withdrawal between groups). 291 included in this substudy. Multi-centre RCT of individuals with Cockcroft–Gault GFR of 15–35 mL/min , Hb 11.0-12.5 g/dL. Systolic/diastolic blood pressure <170/95 mmHg. Recruited between July 2000 and November 2002. Clinical trial followed-up until November 2004. | Anticipated need for renal replacement therapy within 6 months, advanced cardiovascular disease (as defined by a diagnosis of clinically significant valvular disease, congestive heart failure, myocardial infarction, unstable angina, or stroke within the preceding 3 months), non-renal causes of anemia, receipt of blood transfusions within the preceding 3 months, a serum ferritin level of less than 50 ng per milliliter, a C-reactive protein level exceeding 15 mg per liter, and previous treatment with erythropoietin.' | Sudden death, myocardial infarction, acute heart failure, stroke, transient ischemic attack, angina pectoris resulting in hospitalization for 24 hours or more or prolongation of hospitalization, complication of peripheral vascular disease (amputation or necrosis), or cardiac arrhythmia resulting in hospitalization for 24 hours or more.' | Not stated. | Cox regression models. States 'pre-defined' sub-analysis but unclear how. |
| CRIC | ‘3,939 participants with CKD stages 2-4 who enrolled in the Chronic Renal  Insufficiency Cohort (CRIC) between June 2003 and December 2008’. Age 21 to 74 years. 3,904 individuals included in current analysis (see ‘missing data’ column). 7 centres from USA. Follow-up until June 2009, death or voluntary withdrawal from study. | ‘inability to consent, institutionalization, enrolment in other studies, pregnancy, New York Heart Association classes III-IV heart failure, human immunodeficiency virus (HIV) infection, cirrhosis, myeloma, polycystic kidney disease, renal cancer, recent chemotherapy or immunosuppressive therapy, organ transplantation, or prior treatment with dialysis for at least 1 month. | ‘Definite or probable myocardial infarction,  stroke, or peripheral arterial disease’. Outcome adjudicated by ‘blinded reviewers’. | ‘Thirty-five participants  had missing serum bicarbonate levels at baseline and were excluded from this study’. ‘Approximately 9.4% of participants had missing covariate information and were excluded’. Individuals with missing data were ‘not significantly different at baseline’. No specific information on voluntary withdrawal from study in current study. | ‘Multivariable Cox proportional hazards models were used’. ‘Death was treated as a  censoring event when it was not part of the outcome’. Quadratic splines used to explore nonlinearity, but for cardiovascular events a linear relationship was found. Proportional hazard model assumption tested through use of Martingale residuals. ‘interactions by race, diabetes, eGFR, and proteinuria for death and renal outcomes and by diuretic use for cardiovascular outcomes’. Sensitivity analysis performed. Database ‘locked’ for analysis at 30^th^ June 2009. |
| CRISIS | Prospective cohort study. Patients recruited between 1^st^ October 2002 and 31^st^ October 2009. Patients with CKD stages 3-5. eGFR >10 ml/min/1.73m²and <60 ml/min/1.73m². | 1316 patients enrolled in CRISIS cohort. 470 patients included in analysis with OPG, FGF-23 and Fetuin-A measurements. 463 patients included in final analysis due to 7 with incomplete baseline data. | ‘Non-fatal stroke or myocardial infarction, coronary angiogram plus angioplasty or stenting and coronary artery bypass graft surgery. Heart failure defined as left ventricular ejection fraction ≤50% or diastolic dysfunction on echocardiogram or a clinical diagnosis of heart failure with no other alternative cause for symptoms.’ | 7 patients with incomplete baseline data but not included in final analysis. No other mention of incomplete data or loss to follow up. | Multivariate cox proportional hazards regression. |
| Digitalis | Multi-centre RCT of digoxin in chronic systolic and diastolic HF in normal sinus rhythm in 302 centres from 1991 to 1993. eGFR < 60 ml/min per 1.73m2. 7788 patients screened, 2793 in CKD in analysis for this study. | Serum creatinine >2.5 mg/dL, potassium ≥5 mEq/L, | Cardiovascular, and HF hospitalizations.' | Vital status data were complete for 99% of patients during 57 months of follow-up.' 931 had no baseline potassium, 579 excluded due to potassium K ≥5 mEq/L, unclear how many had serum creatinine >2.5 mg/dL | Propensity score matching. Matched Cox regression analysis. |
| Fujita | 404 patients in a 'prospective cohort' of individuals with eGFR lower than 60 ml/min/1.73 m^2^ recruited from February 2009 to September 2010. | Individuals with a 'history of hospitalization for acute coronary syndrome, worsening heart failure (New York Heart Association functional class III or IV), stroke, aortic dissection, or aortic aneurysm within 6 months before enrolment; fever or a sign of infectious diseases.' | CV death, acute coronary syndrome, hospitalization for worsening heart failure, stroke, and dissection of aorta. The diagnosis of acute coronary syndrome was based on the current guidelines. Heart failure was diagnosed based on the Framingham diagnostic criteria. Stroke was defined as clinical signs of focal or global disturbance of cerebral function caused by cerebrovascular damage. Aortic dissection was diagnosed based on computed tomography. The presence of CV events was determined independently by physicians who were blinded.' | No further details on missing data. | Cox proportional hazards regression analysis. All variables with P<0.05 by univariate analysis were included in the adjusted model. Not a pre-specified analysis. |
| Genoa | Multi-centre (2 centres) prospective cohort study. Patients recruited between 1^st^ January 1999 and 31^st^ December 2003. Patients included with non dialysis CKD < 60 ml/min/1.73m,² or GFR 60-90ml/min/1.73m² with proteinuria > 0.3 g/24hr twice with and interval ≥ 3 months. | 693 patients screened. 445 patients included in the final analysis. Patients excluded due to follow up <3 months, no CKD, refusal of ABPM or echocardiography, wall motion abnormalities, inadequate ABPM measurement, office and 24 ABP < 130/80mmHg without antihypertensive treatment, change of antihypertensive treatment prior to study, severe valvular or ischemic heart disease, poor quality echocardiography, atrial fibrillation or bundle branch block, acute GFR change. | Cardiovascular events defined as myocardial infarction, congestive heart failure, stroke, revascularization, peripheral vascular disease, and nontraumatic amputation. Unclear from paper if adjudication team blinded | 74 patients of those screened lost to follow up. 67 of those screened with inadequate or absent ABPM or echocardiography data. | Multivariable cox proportionalhazards regression |
| ICKD | 2 centres prospective cohort recruited from a 'medical centre' and a 'regional hospital'. KDIGO CKD stages 1–5. Recruited between 11/11/2002 and 31/5/2009. | Acute kidney injury 'defined as more than a 50% decrease in eGFR in 3 months'. 356 stages 1-2 CKD patients were excluded. | Cardiovascular events defined as acute coronary syndrome, acute cerebrovascular disease, congestive heart failure, and peripheral arterial occlusion disease and death by aforementioned causes.' | 90 patients lost to follow up. Possible underestimation of cardiovascular events due to patients receiving renal replacement therapy outside of their hospital and subsequently developing cardiovascular events. | Cox proportional hazard model |
| Kaohsiung | 3749 consecutively enrolled outpatient pre-dialysis patients with CKD 3-5 recruited from 11/11/2002 to 31/5/2009. Follow-up until 31/5/2010. 'Most' referred from local medical units or other specialists in the two recruiting hospitals for 'their impairment or progression of renal function'. | 3303 in final analysis. 446 excluded (356 patients CKD stages 1-2, 90 <3 months follow-up). 'significant mitral valve disease, atrial fibrillation, or inadequate image visualization' also exclusion criteria. | Hospitalization for acute coronary syndrome (Deyo’s modified Charlson score, ICD-9 - 410.x–412.x), acute cerebrovascular disease (ICD-9 - 430.x–438.x), congestive heart failure (ICD-9 - 428.x), and peripheral arterial occlusion disease (ICD-9 443.9, 441.x, 785.4, V43.4, procedure 38.48) and death by aforementioned cause'. 'Ascertained by reviewing charts' | 90 patients lost to follow-up within 3 months. 193 patients included had more than 20% missing data. | Cox proportional hazard model. Significant variables (<0.05) in univariate analysis were selected for multivariate forward analysis. Not pre-specified analysis |
| Kyushu | Prospective observational study of 320 consecutive admitted to single centre for 'evaluation of and education about CKD' from January 2005 to September 2012. Japanese patients with CKD stages 3-5, eGFR < 60 ml/min per 1.73m^2^. No discussion of how many screened for study. | Malignancy or history of PAD. | PCI/CABG for IHD, CHF, CVD (brain infarction and haemorrhage), carotid endartectomy, percutaneous transcatheter angioplasty, lower limb amputation, bypass for PAD, dissecting aneurysm for thoracic and or abdominal aorta, rupture of aneurysm, PE, sudden death' | No further details on missing data. | Cox proportional hazard model. Significant variable in univariate analysis were selected for multivariate forward analysis. Unclear if pre-specified analysis |
| Leuven | Prevalent CKD patients seen at a single centre nephrology outpatient clinic. Recruited between November 2005 and September 2006. Followed up until 31/12/2010. CKD stages 1 to 5. | 548 individuals eligible, 49 excluded as not eligible for study. 476 out of 499 analysed in this study. 13 FSGS patients and 10 patients without suPAR measurement excluded. | ‘Composite of death from cardiac causes, nonlethal MI, myocardial ischemia, coronary intervention, ischemic stroke, or new-onset peripheral vascular disease’. ‘Cases of unobserved sudden death were considered cardiovascular death only when other potential causes could be excluded’. ‘‘End points were prospectively recorded and coded, blinded from clinical and biochemical data’. | ‘If information [regarding outcome] could not be obtained, the patient was assumed to be lost to follow-up starting from the date of the last actual visit’. Paper does not stated how many this occurred to. | Cox proportional hazard model |
| Madrid | 135 patients from renal clinic between January and May 2007 screened. eGFR lower than 60 ml/min; stable clinical condition in terms of no hospitalizations nor cardiovascular events within the 3 months before screening; and 'stable renal function' (baseline serum creatinine had not increased by 50% in the 3 months before screening). 113 included in study. | History of allopurinol intolerance or already on allopurinol treatment, active infections or inflammatory diseases, HIV infection, chronic hepatopathy, patients receiving immunosuppression. 22 excluded as eGFR >=60. 98 completed study - 2 started HD, 9 lost to follow up, 2 deaths, 2 due to 'minor adverse events' | Myocardial infarction, coronary revascularization, or angina pectoris. Congestive heart failure (CHF) was diagnosed by x-ray examination (pulmonary edema) and echocardiogram with left ventricular dysfunction. This diagnostic was considered as the patients were symptomatic and in New York Heart Association (NYHA) class II to IV with a left ventricular ejection fraction <45%. Cerebrovascular disease was established if the patient had a history of transient ischemic attacks, whenever stroke could be verified by computer tomography or carotid artery stenosis 70% could be verified by doppler ultrasound. Peripheral vascular disease was diagnosed by intermittent claudication, stenosis of the major arteries of the lower limbs angiographically or sonographically proven, and the presence of ulcers caused for atheroesclerotic disease or by surgery was used for diagnosis. Each event was reviewed by physicians. This information always included study hospitalization records and in the case of an out-of hospital death, family members were interviewed by telephone to better ascertain the circumstances surrounding death. Clinicians blinded during assessment.' | 9 lost to follow-up | Cox proportional hazard models. |
| MAURO | 755 patient with CKD stages 2-5 from 22 southern Italy nephrology units recruited from October 2005 to September 2008. Aged 18-75 and in 'stable clinical condition'. | Rapidly evolving renal disease, kidney transplant, acute intercurrent infections or acute inflammatory processes, pregnancy, cancer, or diseases in the terminal phase.' Creatinine >1.5-4.0 mg/dL in men and >1.3-3.5 mg/dL in women. Pregnancy. | Myocardial infarction, documented by electrocardiography and biomarkers of myocardial injury; heart failure, defined as dyspnea in addition to two of the following conditions: raised jugular pressure, bi-basilar crackles, pulmonary venous hypertension, or interstitial edema on chest radiography requiring hospitalization; electrocardiography documented arrhythmia; stroke; peripheral vascular disease; and major arterial or venous thrombotic episodes' | No mention in current paper. | Cox regression model. |
| Naples | Multicenter prospective cohort study of consecutive patients attending 4 outpatient nephrology clinics in Italy between 1/1/2003 and 31/12/2005'. Follow-up until 31/12/12. | 530 screened, 472 eligible, 436 included. Excluded if missed more than 20% of treatment. From another publication from the same cohort 'We included 459 of 530 eligible patients, reasons for exclusion were inadequate ABPM recordings (n = 35), change of antihypertensive therapy 2 weeks before the study (n = 24), atrial fibrillation (n = 8), and a GFR change of more than 30% (n = 4). | Cardiovascular death or nonfatal cardiovascular event that required hospital stay (myocardial infarction, congestive heart failure, stroke, revascularization, peripheral vascular disease, and non-traumatic amputation), whichever occurred first. Hospital records were obtained to establish diagnosis' | 23 patients excluded as lost to follow-up after initial visit. | Cox proportional hazards model, stratified by center. |
| OSERCE-2 | Observational prospective study of 39 nephrology centers of CKD stages 3–5 enrolled. Consecutive recruitment by including the first 20 adult patients. 742 baseline patients, only those with radiological data included in analysis (77%). Study from April 2009 May 2012. | Acute renal failure, serious illness that presupposed a life expectancy of <12 months, and hospital admission during the month before inclusion. | Cardiovascular hospitalization' | 20 unexplained deaths. | Cox proportional hazards model. |
| Pravastatin | 3 RCTs (WOSCOPS, CARE & LIPID). All double blinded 40mg pravastatin versus placebo for approximately 5 years. CKD defined as eGFR <60 or 60-89.9 with at least proteinuria. Initially, 19,737 patients, 4099 (20.8%) had CKD but not DM, 571 (2.9%) had CKD and DM. | Excluded based on serum creatinine greater than certain level (WOSCOPS >1.7mg/dL, CARE >2.5mg/dL, LIPID >4.5mg/dL). | Coronary heart disease death, nonfatal MI, or coronary revascularization (coronary artery bypass grafting or percutaneous transluminal coronary angioplasty). Secondary outcome - the time to an expanded composite cardiovascular outcome (first occurrence of coronary heart disease death, nonfatal MI, coronary revascularization, or nonfatal stroke)', blinded observers. | Not reported in current combined analysis manuscript | Proportional hazards regression models. |
| RRI | 305 subjects included for analysis with 24 hour Holter data. From the main RRI-CKD trial 627 were enrolled between 1/1/2000 and 31/12/2002 of which 408 were alive at 1/1/2003. For this sub-analysis, 149 recruited who were alive at end of main trial. An additional 199 individuals were recruited for this subanalysis. 43 declined to have Holter monitor. Follow-up until 31/12/2006. | Transient renal impairment prior to enrolment (eGFR on two occasions at least 1 month apart). Those recruited were ‘significantly healthier than those who did not' (younger, higher mean eGFR, less DM, less CVD history). Nitro-glycerine patch, defibrillator, active pacing or with allergy to electrode adhesive material) were excluded. | CAD, cerebrovascular disease, peripheral arterial disease, CHF and cardiac arrest. All outcomes were ascertained on an ongoing basis by study coordinators from regular review of electronic health records, direct patient contact in clinic and periodic telephone communication. | Not reported. | Cox regression. |
| TREAT | 4038 patients with T2DM, CKD and anaemia, previously enrolled into the TREAT randomised controlled trial. 623 sites in 24 countries, from Aug 2004 to Dec 2007. | Exclusions for ‘uncontrolled HTN, previous renal transplant or scheduled for live donor transplant, current IV Abx/chemotherapy/radiotherapy, cancer, HIV, active haematological disease / bleeding, pregnancy, recent MACE/seizure/surgery (last 12 weeks)’. 4047 patients enrolled, 9 excluded (not for clinical reasons). | ‘MI, stroke, ESRD, and the composite of cardiovascular death, MI or hospitalization due to ischemia, heart failure or stroke.’ Definitions reported in main trial manuscript. End points adjudicated by a blinded clinical end point committee. | Outcomes reported for all 4038 patients. No loss to follow-up reported in original study. | Cox proportional hazards models. |

**Supporting Table E** – List of all 66 risk factors identified

| **Variable** | **Study** | **Details** | **Included in Systematic Review** | **Rationale** | **Main Manuscript Reference Number or Reference if Different to Main Manuscript** |
| --- | --- | --- | --- | --- | --- |
| Adragao score | OSERCE-2 | based on presence of calcification on plain radiographic films of pelvis and hands | No | Not routinely collected | 37 |
| Age | See forest plot | at baseline | Yes | - | See forest plot |
| Albumin | See forest plot | serum | Yes | - | See forest plot |
| Ankle–brachial pressure index | Kyushu, OSERCE-2 | - | No | Not routinely collected | 32,37 |
| Baseline NT-proBNP | CREATE | serum | No | Not routinely collected | 27 |
| Beck Depression Index II | AASK - cohort | multiple-choice self-reporting questionnaire for depression | No | Not routinely collected | Fischer M et al, Kidney International; (2011) 80:670-678 |
| Beta-2 microglobulin | CRIC | serum | No | Not routinely collected | Foster MC et al, Am J Kidney Dis. 68(1):68-76. |
| Beta-trace protein | CRIC | serum | No | Not routinely collected | Foster MC et al, Am J Kidney Dis. 68(1):68-76. |
| Bicarbonate | CRIC | serum | Yes | - | Dobre M et al, American Journal of Kidney Disease; 2013; 62(4):670-678 |
| Body Mass Index | See forest plot | kg/m^2^ | Yes | - | See forest plot |
| Calcium | See forest plot | serum | Yes | - | See forest plot |
| Cardiovascular disease | See forest plot | diagnosis at baseline | Yes | - | 30 |
| Cholesterol to HDL ratio | ICKD | serum | Yes | - | 30 |
| Clinic versus ambulatory blood pressure | Minutolo *et al,,*  *Kushiro et al* | comparison of clinic versus ambulatory blood pressure to categorise patients | No | Ambulatory blood pressure not routinely collected | Minutolo R et al, American Journal of Kidney Disease; 2014; 64(5):744-752  Kushiro T et al, Hypertension Research (2017) 40, 87–95 |
| Congestive heart failure | CREATE | diagnosis at baseline | Yes | - | 27 |
| C-reactive protein | Kyushu, Madrid, Haryana, Leuven | serum | No | Not routinely collected for purpose of CV risk assessment | 32,33,34 Haryana - Nand N et al, Journal, Indian Academy of Clinical Medicine; 2009; 10(1 & 2): 18-22 |
| Diabetes mellitus | See forest plot | diabetes mellitus at baseline | Yes | - | See forest plot |
| Diabetic nephropathy | CREATE | diabetic nephropathy at baseline | No | Not routinely collected | 27 |
| Diastolic blood pressure | See forest plot | clinic based | Yes | - | See forest plot |
| Degree (high school) | AASK - RCT | education | No | Not routinely collected | 24 |
| Dyslipidaemia | Kyushu | multiple categories | No | Multiple categorical variables | 32 |
| Electrocardiogram | AASK - RCT | normal' versus 'abnormal' | No | Definition unclear from reference | 24 |
| Fetuin-A | CRISIS | serum | No | Not routinely collected | 30 |
| Fibroblast growth factor-23 | CRISIS | serum | No | Not routinely collected | 30 |
| Gender | See forest plot | - | Yes | - | See forest plot |
| Hemoglobin | See forest plot | serum | Yes | - | See forest plot |
| High density lipoprotein | ICKD | serum | Yes | - | 30 |
| High sensitivity C-reactive protein | Fujita, Ankara | serum | No | Not routinely collected | 25,29 |
| Homocysteine | HOPE-2 | serum | No | Not routinely collected | Mann J et al, Nephrology Dialysis Transplantation; 2008; 23: 645–653 |
| Hydration status | Tsai *et al* | bioelectric impedence spectroscopy measured at baseline | No | Not routinely collected | Tsai Y-C et al, Clinical Journal of the American Society of Nephrology; 2015; 10: 39–46 |
| IL-33 | Ankara | serum | No | Not routinely collected | Gungor O et al, PLoS ONE 12  (6): e0178939 |
| Income | AASK - RCT | self-reported monetary income at baseline | No | Not routinely collected | 24 |
| Ischemic Heart Disease | See forest plot | diagnosis at baseline | Yes | - | See forest plot |
| Kauppila score | OSERCE-2 | based on presence of calcification on plain radiographic films of lumbar spine | No | Not routinely collected | 37 |
| Left ventricular end-diastolic volume | Kaohsiung | echocardiogram | No | Not routinely collected | 31 |
| Left ventricular hypertrophy | AASK - RCT | echocardiogram | Yes | - | 24 |
| Left ventricular mass index | CREATE | echocardiogram | No | Not routinely collected | 27 |
| Low density lipoprotein | See forest plot | serum | Yes | - | See forest plot |
| Mean arterial pressure | AASK - RCT | clinic based | Yes | Not routinely collected | 24 |
| Metabolic Syndrome | MESA | as a whole diagnosis (not the presence of individual parts such as HTN, DM etc) | No | Not routinely collected, categorical variables | Agarwal S et al, Cardiology Research and Practice; 2012; Article ID 806102 |
| Non-HDL cholesterol | ICKD, AASK - RCT | serum | Yes | - | - |
| Osteoprotegerin | CRISIS | serum | No | Not routinely collected | 30 |
| Parathyroid Hormone | CARE FOR HOMe | serum | Yes | - | - |
| Peripheral Vascular Disease | CREATE | diagnosis at baseline | No | Definition unclear from reference | 27 |
| Phosphate | See forest plot | serum | Yes | - | See forest plot |
| Potassium | Digitalis | serum | No | Unable to compare due to multiple serum categories | 28 |
| Prolactin | Heraklion | serum | No | Not routinely collected | Carrrero J et al; Clinical Journal of the American Society of Nephrology; 2012; 7: 207–215 |
| Pseudoresistant hypertension | Naples | normal ambulatory blood pressure but raised clinic blood pressure | No | Not routinely collected | 36 |
| Pulmonary hypertension | CRIC | echocardiogram | Yes | - | 29 |
| Pulse pressure | Kyushu | clinical based | Yes | - | See forest plot |
| Root mean square of the successive differences of heart rate | CRIC | - | No | Not routinely collected | Drawz P et al; American Journal of Nephrology;2013;38(6): 517–528 |
| Sodium | CanPREDDICT | serum | Yes | - | 27 |
| Sodium | CRIC | urine (24 hour collection) | No | Not routinely collected | Mills KT et al, JAMA. 2016;315(20):2200-2210 |
| Sodium to potassium ratio | AASK - RCT | urine | No | Not routinely collected | 24 |
| Systolic blood pressure | See forest plot | clinic based | Yes | - | See forest plot |
| Systolic blood pressure visit-to-visit variability | AASK - RCT | - | No | Not routinely collected | 24 |
| Standard deviation of NN intervals for heart rate variability | CRIC | - | No | Not routinely collected | Drawz P et al; American Journal of Nephrology;2013;38(6): 517–528 |
| Trimethylamine N-oxide | CanPREDDICT | serum | No | Not routinely collected | 27 |
| Urea | AASK - RCT | serum | Yes | - | 24 |
| Smoking | See forest plot | smoker at baseline versus non-smoker | Yes | - | See forest plot |
| ST2 | Ankara | serum | No | Not routinely collected | Gungor O et al, PLoS ONE 12  (6): e0178939 |
| Sustained hypertension | Naples | raised ambulatory blood pressure but normal clinic blood pressure | No | Not routinely collected | 36 |
| Total cholesterol | See forest plot | serum | Yes | - | See forest plot |
| True resistant hypertension | Naples | raised ambulatory and clinic blood pressure | No | Not routinely collected | 36 |
| Urate | See forest plot | serum | Yes | - | See forest plot |
| Years with hypertension | AASK - RCT | years of diagnosis at baseline | No | Not routinely collected | 24 |

**Supporting Figures** – Forest Plots for all Risk Factors Meta-analysed

All variables and units are listed as in table 3 of the main manuscript.

**A -Male**

**
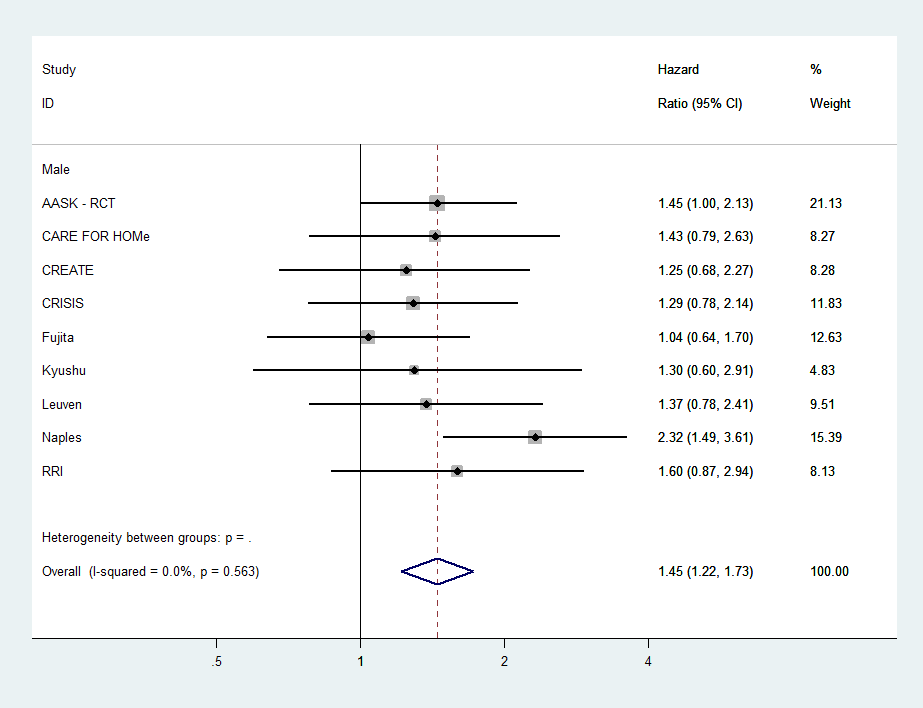
**

**B - Age**

**
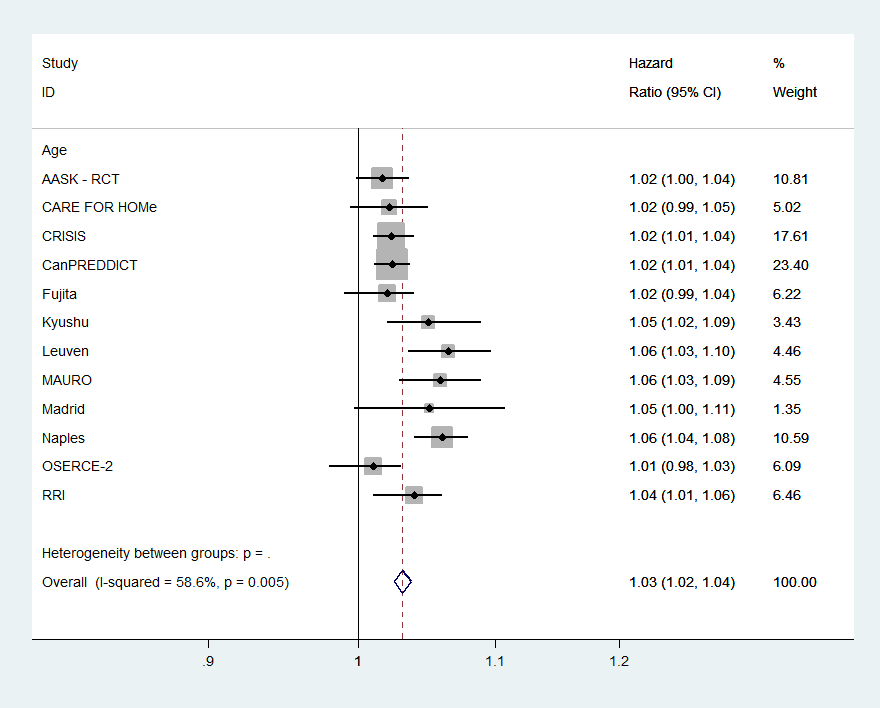
**

**C - Smoking**

**
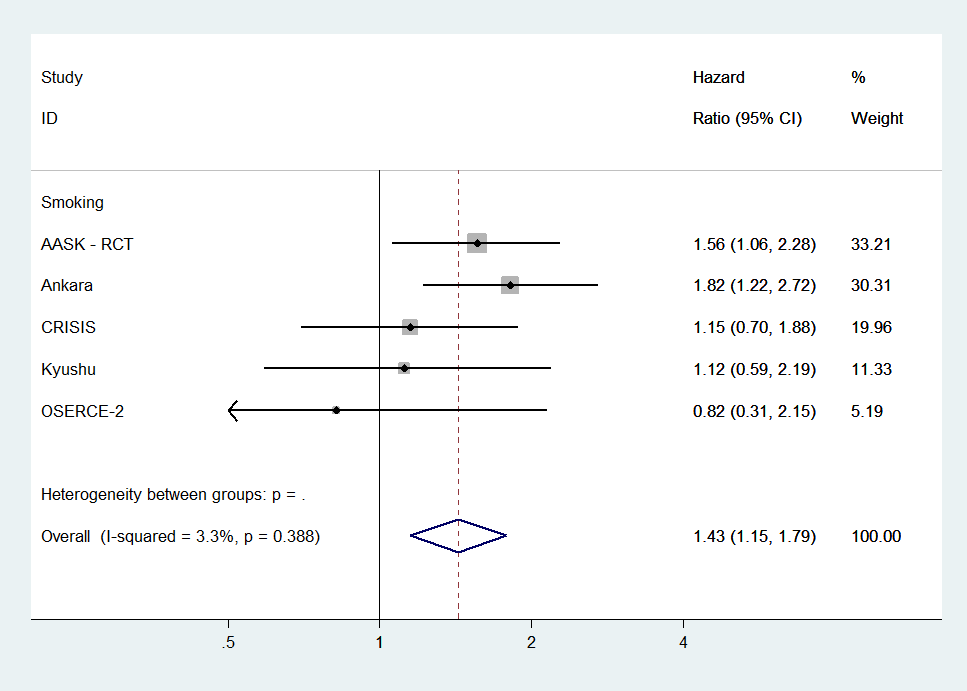
**

**D - Body Mass Index**

**
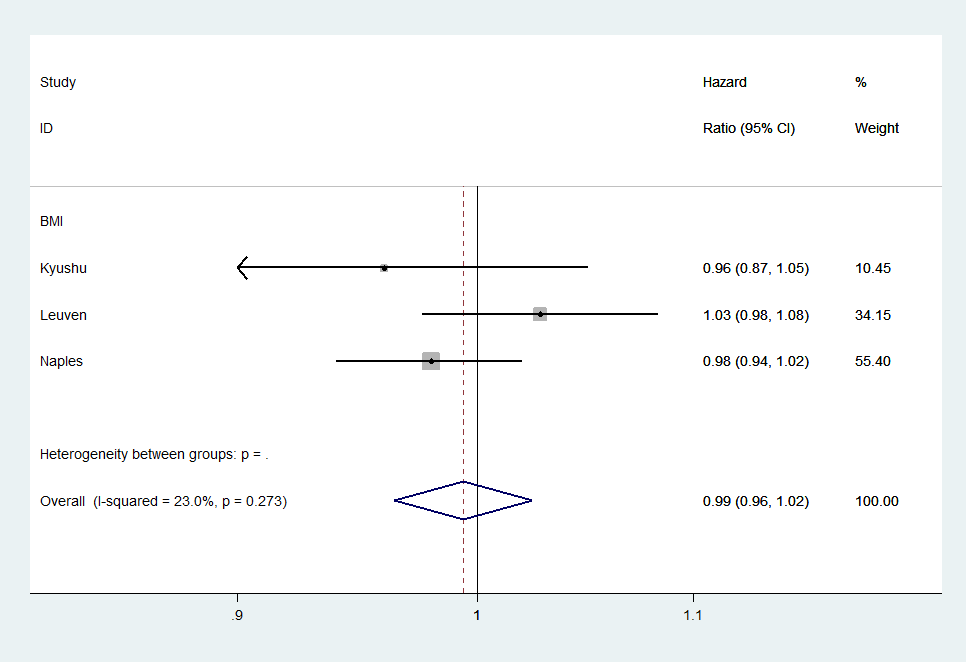
**

**E - Cardiovascular Disease**

**
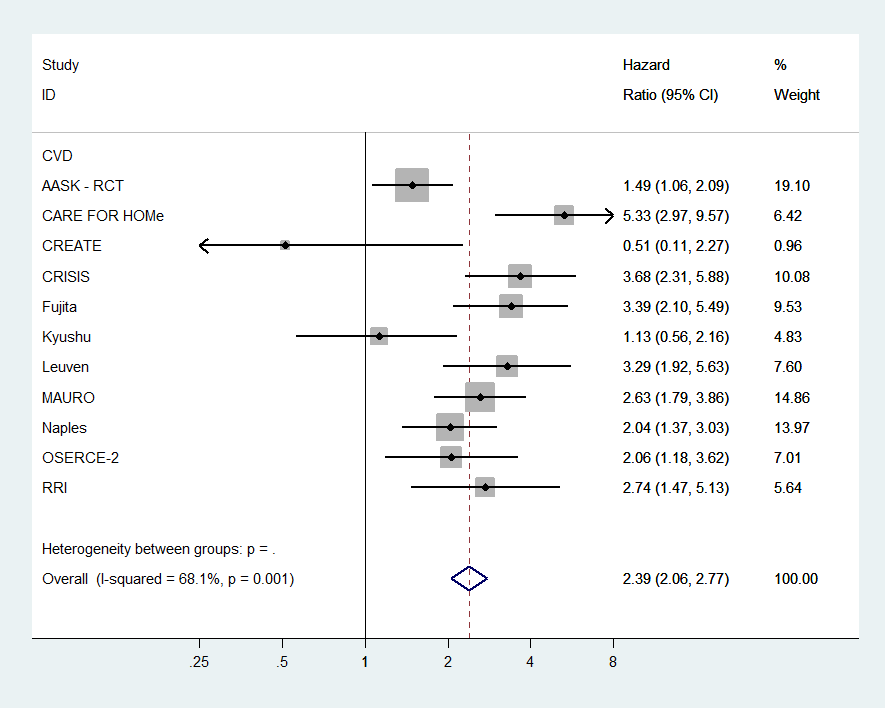
**

**F - Ischaemic Heart Disease**

**
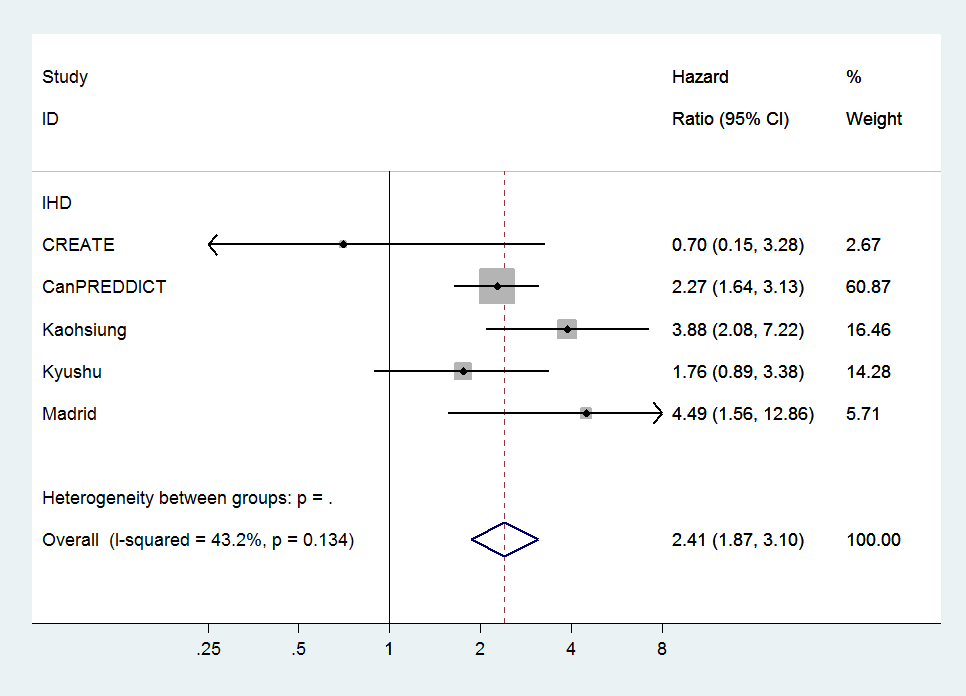
**

**G - Congestive Heart Failure**

**
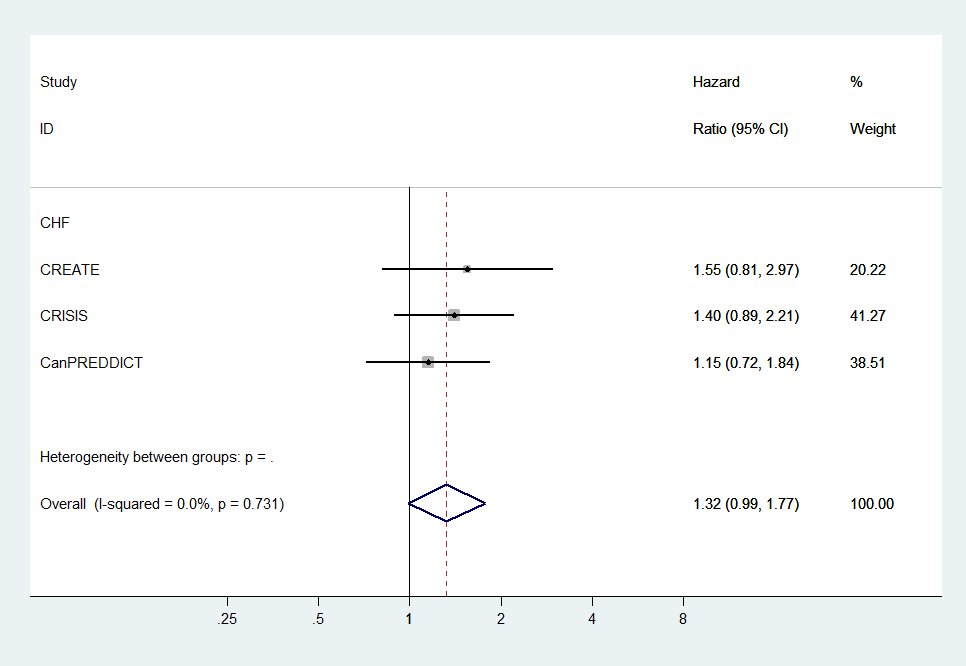
**

**H - Diabetes Mellitus**

**
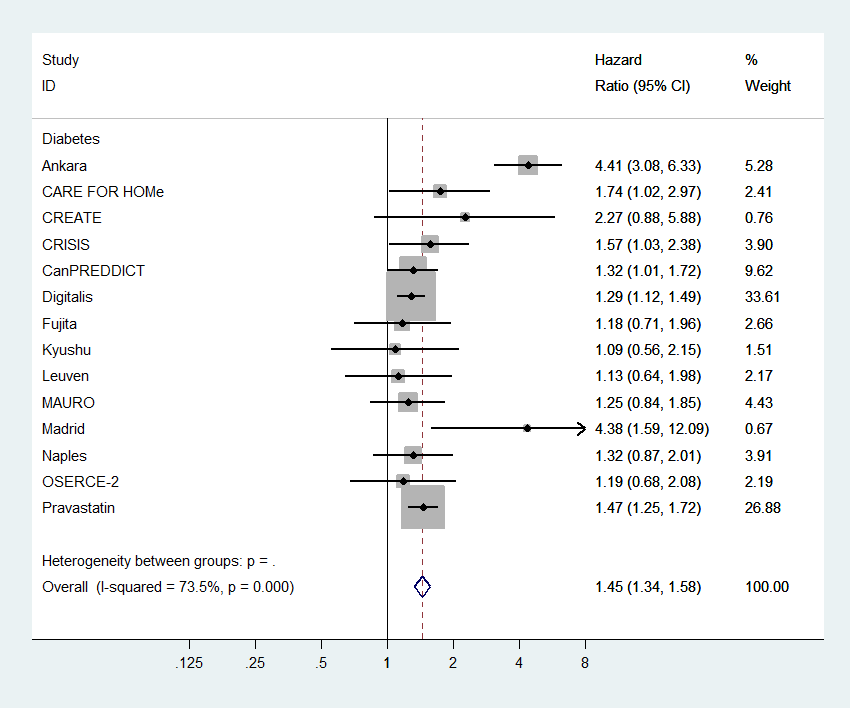
**

**I - Systolic Blood Pressure**

**
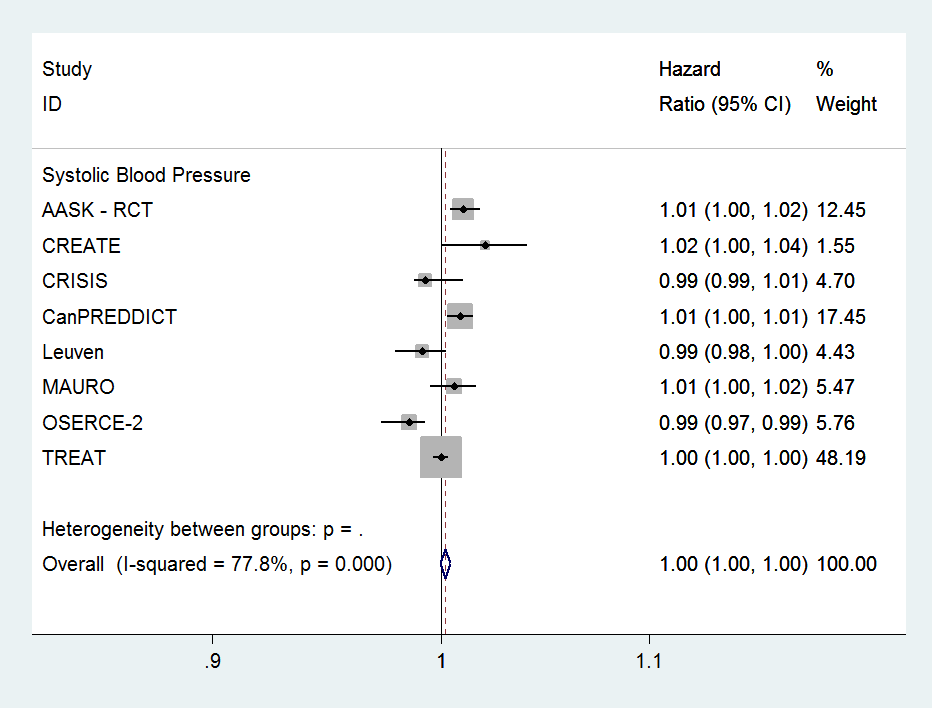
**

**J - Diastolic Blood Pressure**

**
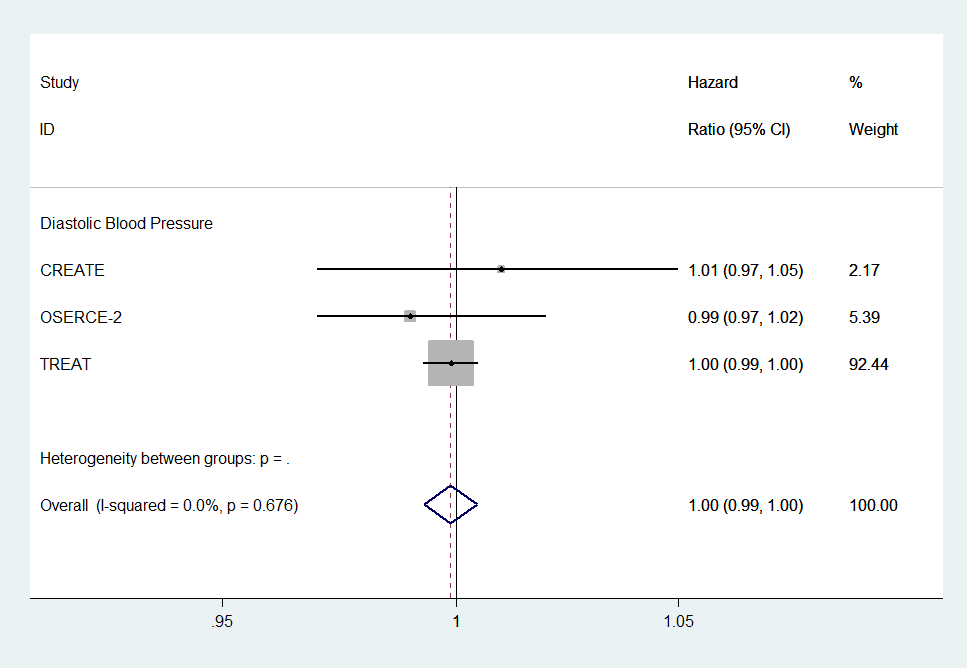
**

**K - Pulse Pressure**

**
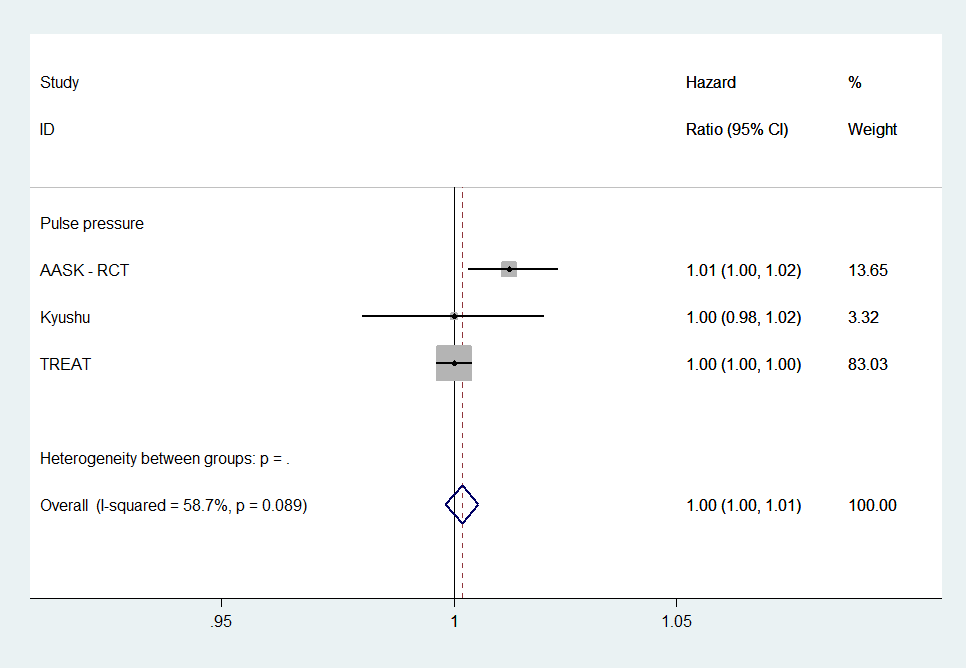
**

**L - Low Density Lipoprotein Cholesterol**

**
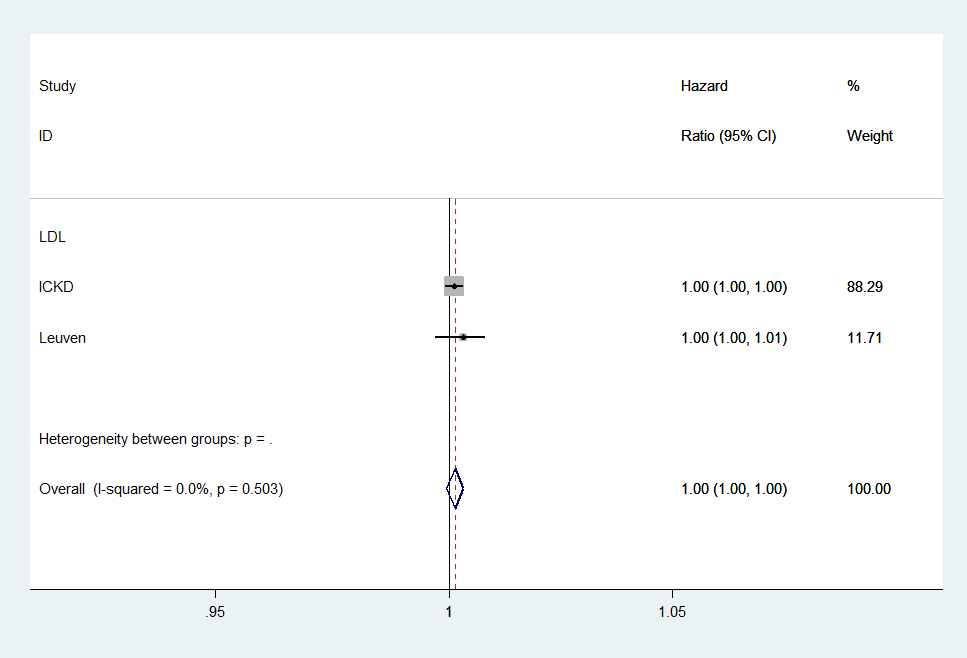
**

**M - Non-high Density Lipoprotein Cholesterol**

**
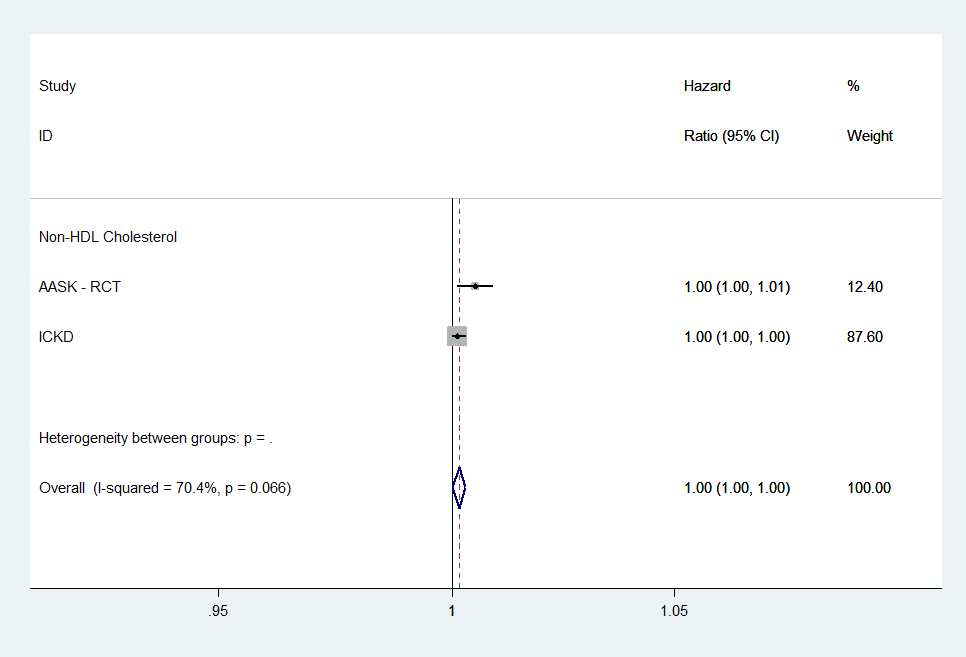
**

**N - Total Cholesterol**

**
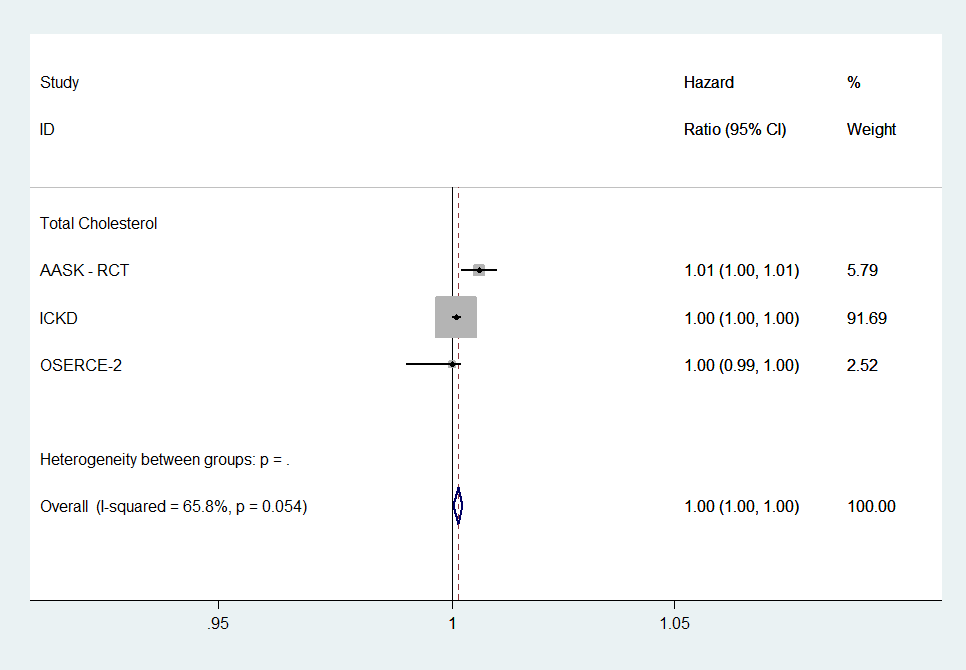
**

|  |  |  |  |  |  |
| --- | --- | --- | --- | --- | --- |
|  |  |  |  |  |  |
|  |  |  |  |  |  |
|  |  |  |  |  |  |
|  |  |  |  |  |  |
|  |  |  |  |  |  |
|  |  |  |  |  |  |
|  |  |  |  |  |  |
|  |  |  |  |  |  |
|  |  |  |  |  |  |
|  |  |  |  |  |  |
|  |  |  |  |  |  |
|  |  |  |  |  |  |
|  |  |  |  |  |  |
|  |  |  |  |  |  |
|  |  |  |  |  |  |
|  |  |  |  |  |  |
|  |  |  |  |  |  |
|  |  |  |  |  |  |
|  |  |  |  |  |  |
|  |  |  |  |  |  |
|  |  |  |  |  |  |
